# Lateralized antennal control of aggression and sex differences in red mason bees, *Osmia bicornis*

**DOI:** 10.1038/srep29411

**Published:** 2016-07-08

**Authors:** L. J. Rogers, E. Frasnelli, E. Versace

**Affiliations:** 1 Centre for Mind/Brain Sciences, University of Trento, Piazza della Manifattura 1, I-38068 Rovereto, Italy; 2 School of Science and Technology, University of New England, Armidale, NSW 2351, Australia; 3 Psychology, College of Life and Environmental Sciences, University of Exeter, Exeter EX4 4QG, UK

## Abstract

Comparison of lateralization in social and non-social bees tests the hypothesis that population-level, directional asymmetry has evolved as an adjunct to social behaviour. Previous research has supported this hypothesis: directional bias of antennal use in responding to odours and learning to associate odours with a food reward is absent in species that feed individually, such as mason bees, whereas it is clearly present in eusocial honeybees and stingless bees. Here we report that, when mason bees engage in agonistic interactions, a species-typical interactive behaviour, they do exhibit a directional bias according to which antenna is available to be used. Aggression was significantly higher in dyads using only their left antennae (LL) than it was in those using only their right antennae (RR). This asymmetry was found in both males and females but it was stronger in females. LL dyads of a male and a female spent significantly more time together than did other dyadic combinations. No asymmetry was present in non-aggressive contacts, latency to first contact or body wiping. Hence, population-level lateralization is present only for social interactions common and frequent in the species’ natural behaviour. This leads to a refinement of the hypothesis linking directional lateralization to social behaviour.

Research on vertebrate species has shown that it is beneficial to have a lateralized brain so that information can be processed differently and simultaneously on the left and right sides. For example, chicks with lateralized processing of visual information can attend to two tasks at the same time (viz., searching for grains of food while monitoring overhead for a potential predator), whereas chicks not lateralized in this way perform poorly on both aspects of the task^1^. A similar result has also been found in a comparison of lateralized and non-lateralized fish^2^. Pigeons, also, are more efficient at finding grain amongst non-food items (grit) if they are more strongly lateralized^3^. Hence, cognitive/behavioural efficiency can be enhanced by having a lateralized brain.

It remains less clear, however, what advantage might ensue from the majority of individuals in a population or a species displaying the same direction of lateralization when increased neural efficiency and capacity could be achieved by lateralization at the individual level alone. Indeed, since the left eye detects predators and controls escape and attack responses, it seems disadvantageous for the majority of individuals to have the same direction of lateralization: predators could take advantage of this bias by approaching their prey on the right. Nevertheless, evidence of a population directional bias in anti-predator responses has been found also in sheep^4^ and in shoaling fish^5^. Another potential disadvantage of a population-level bias concerns foraging behaviour: some prey or other food items on the left side could be overlooked since it is the right eye (and left hemisphere) that is specialized to pursue prey and direct feeding responses (summarized in ref. 6).

To explain the widespread existence of population-level biases, it has been hypothesized that functional lateralization at the population level might have arisen as an Evolutionary Stable Strategy when lateralized individuals interact^7^,^8^. In other words, when individually asymmetric organisms must interact with conspecifics and coordinate their activities, asymmetry aligns in the majority of individuals in a population (i.e., directionality)^9^,^10^. If so, population-level lateralization should be more common for social interactions frequently encountered during the course of evolution. With the discovery of functional lateralization in invertebrate species (see ref. 11), and bees in particular^12^, it has become possible to test this hypothesis. Different species of bees have been categorized as having different degrees of sociality^13^,^14^, from solitary to subsocial, quasisocial and eusocial, mainly according to reproductive strategies and behaviour such as sharing a nest, cooperation in parental care, role division in reproduction between sterile members of the colony and reproducers, and an overlap of generations in the colony. However, other types of interactions, such as aggressive and sexual displays, have not been taken into account in the categorisation of social behaviour in bees.

Some evidence in support of the hypothesis concerning population-level lateralization has already been obtained by comparing lateralization in the strongly eusocial honeybee, *Apis mellifera*, in which workers interact during foraging and feeding, with lateralization in a species of bee considered to be solitary and that forages and feeds individually, the mason bee *Osmia bicornis*, known previously as *Osmia rufa* (the red mason bee, hereafter referred to in this paper simply as ‘mason bee’). The studies showed that, whereas worker honeybees exhibit a strong population bias for using a preferred antenna in recalling a learnt memory of an association between a particular odour and a food reward^15^, this is not the case for mason bees^16^. A crucial difference in the ecology of these species is that honeybees, but not mason bees, exchange information based on memories about food. Furthermore, electroantennographic responses to an alarm pheromone and a volatile floral compound were found to be lateralized in worker honeybees but not in female mason bees^16^. Consistent with this, seasonally eusocial bumblebees also have a population-level lateralization of antennal use in associating an odour with a food reward^17^ and three species of social Australian stingless bees, the lineage of which emerged much earlier than that of honeybees^18^,^19^, also present the same population-level lateralization as do honeybees^20^.

These results are consistent with the hypothesis outlined above^7^, that social interactions experienced in the course of evolution might lead to evolution of directional lateralization at the population-level, or might co-evolve with it. In fact, although mason bees are solitary in the sense that every female is fertile and makes her own nest^21^ and they forage as individuals without social contact, the species is not entirely devoid of social behaviour. Males emerge from their nests before the females and tend to cluster at the nest site and on flowers while they await the emergence of the females. During this time and during mating itself at least some degree of social behaviour occurs (e.g., female mason bees often compete for nest sites and this involves agonistic interactions^22^). The question we addressed here was, therefore, whether mason bees would exhibit lateralization in performing social interactions, particularly in competition between females.

Previously we tested dyads of honeybees in Petri dishes, and demonstrated lateralization of social behaviour^23^. In encounters between dyads of honeybees in which both bees had only the right antennae (the left ones having been removed) appropriate social behaviour was observed: if both honeybees were from the same hive, contact was made after a short latency, proboscis extension (positive) responses were high and C-responses (aggressive responses used in stinging) were almost absent, whereas latency to contact was longer and C-responses were common when each bee was from a different hive. In contrast, when both bees had only the left antennae, C-responses between bees from the same hive were high and proboscis extension responses were low, and C-responses were lower for dyads from different hives than they were for dyads from the same hive. The latter is inappropriate social behaviour, because it shows no adjustment of agonistic behaviour as a function of the social context. In summary, worker honeybees display asymmetry of antennal use in important aspects of social behaviour and they do so with a population-level bias.

In the present study we were interested to see whether mason bees might show population level lateralization of any aspect of behaviour when tested in a similar apparatus as used in^23^ and with one or the other antenna removed (i.e. to test whether population level lateralization is behaviour- rather than species- specific). As mason bees are either male or female, we tested dyads of females, dyads of males and dyads of a male and a female. This experimental design permitted us to investigate possible lateralized responses that might differ between males and females, which we considered might be important since male-female differences in patterns of agonistic behaviour have been found in other insects, such as the fruitfly^24^. A difference in size was another reason for expecting possible differences in behaviour: female mason bees are considerably larger than males (Fig. 1).

We adopted the method of testing immediately after surgical removal of one or other of the antennae because (1) this method has been used successfully to reveal lateralization in other experiments with bees and (2), although the method of coating one or the other antenna with a silicone compound has been used to block sensory perception and reveal lateralization in harnessed bees^15^,^25^, it cannot be used effectively in free-moving bees since they persistently try to remove the silicone rather than perform other behaviour. Removal of an antenna does not lead to repetitive wiping responses of the head region that interfere with interactions between bees. We also preferred to test the immediate effects on laterality after removal of an antenna rather than any longer-term effects that may involve neural adjustments.

## Results

**Figure 2:**
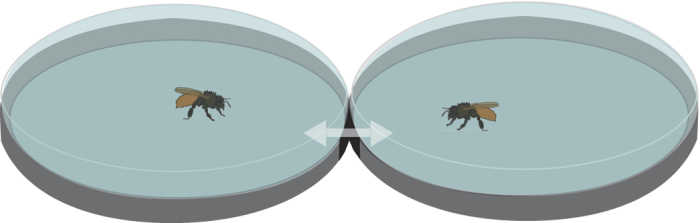
The test apparatus consisted of two adjacent Petri dishes. When the experimenter aligned the small openings cut on the side of each dish (coloured black in the figure), the test period started. The arrows indicate the possibility for the bees to move from one dish to another.

We scored different behaviours of dyads of mason bees located in two interconnected dishes (see Fig. 2). Dyads differed in Sex (male + male; female + female; male + female pairs) and antennae in use (left-left, right-right, both antennae-both antennae, left-right).

### Latency to first contact

**Figure 3:**
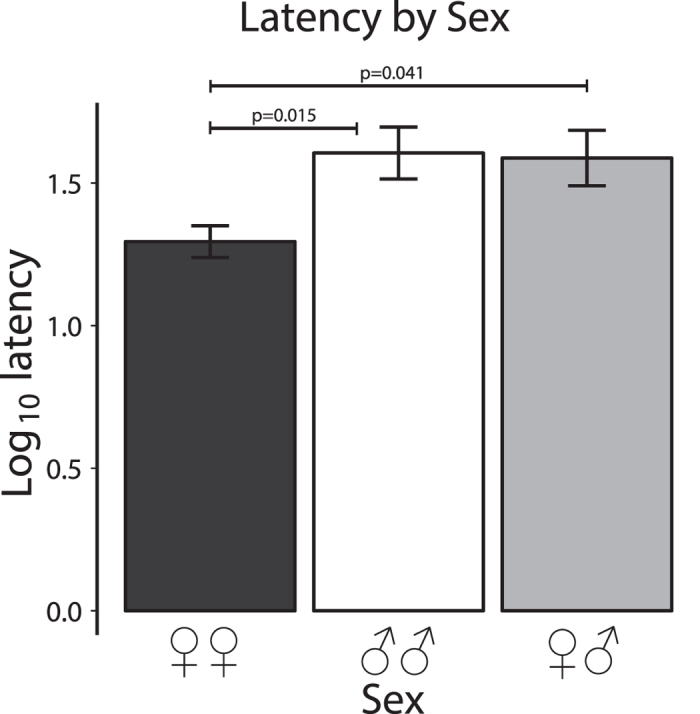
Latency to first contact. Since there was no effect of dyad-type, only data showing the sex difference is plotted. Note the significantly shorter latency scores in dyads of 2 females (black bar) compared to dyads of 2 males (white bar) and male plus female dyads (grey bar). Means (+/−1 standard error) of the log10 scores are plotted.

These results are presented in Fig. 3. Analysis of the log_10_-transformed data revealed a significant effect of Sex (F_2,104_ = 4.902, p = 0.009): female-female pairs contacted after shorter latency than did male-male pairs (p = 0.015) and female-male pairs (p = 0.041). However, there was no significant effect of Dyad-type (F_3,104_ = 0.897, p = 0.445) and no significant interaction between these two factors (F_5,104_ = 1.357, p = 0.247).

### Number of contacts without aggression

**Figure 4:**
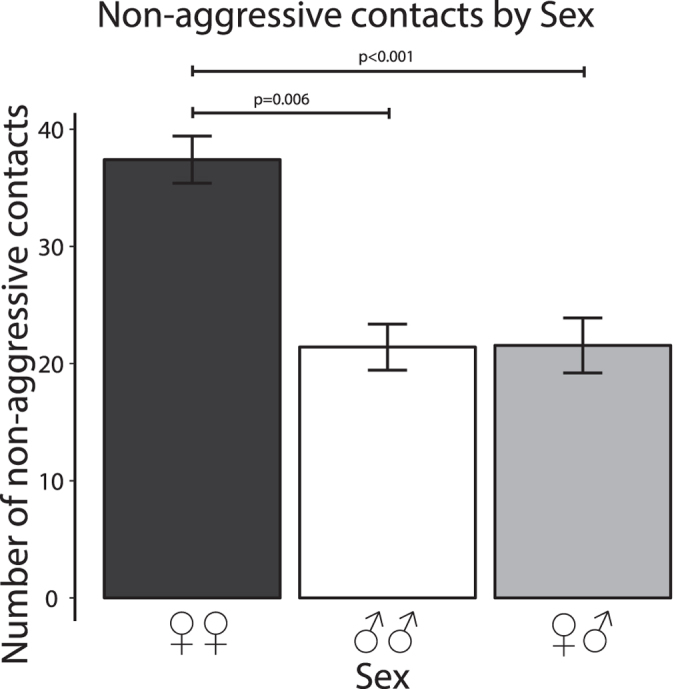
Number of contacts without aggression. As for Fig. 3, these data are plotted to show the sex difference only because there was no significant effect of dyad type. Dyads of 2 females contacted each other fewer times than did dyads of two males or mixed-sex dyads. The labelling is as in Fig. 3.

These data were normally distributed (Shapiro test, p = 0.151). As for latency, the only significant effect was for Sex (F_2,104_ = 19.771, p < 0.001): dyads of females made significantly more contacts than did dyads of males (p = 0.006) or dyads of male + female (p < 0.001), see Fig. 4. There was no significant effect of Dyad type (F_3,104_ = 0.568, p = 0.637) and no significant interaction between these two factors (F_5,104_ = 1.216, p = 0.307).

### Number of aggressive interactions

**Figure 5:**
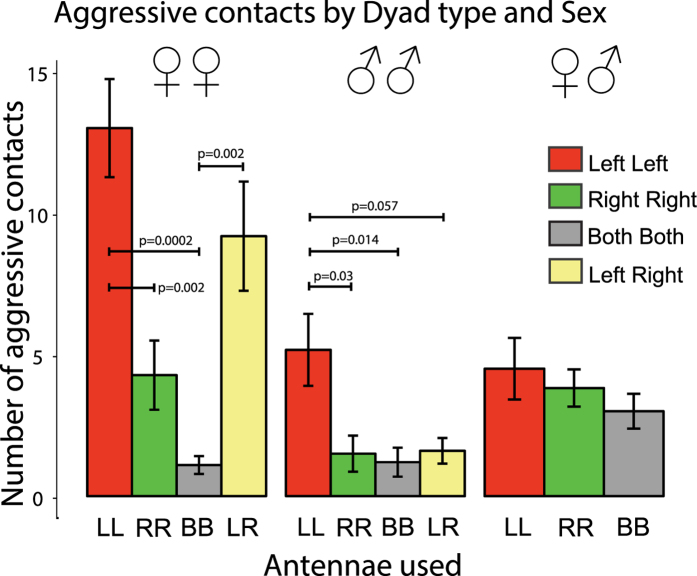
Aggressive contacts, in which one bee rammed the other, which responded by chaotic buzzing while overturned. Means +/− 1 standard error are plotted for the following dyads: both bees using left antennae, LL (red bars), both bees using right antennae, RR (green bars), both bees intact, BB (grey bars) and one bee using the left antenna and the other the right antenna, LR (yellow bars). The sex combinations are plotted separately: from left to right, 2 females, 2 males and male plus female. Note, in particular, the significant difference between LL and RR in both females and males.

Non-parametric analysis of these data revealed an overall effect of Dyad-type (H_3_ = 20.95, p = 0.001) and also one of Sex (H_2_ = 13.30, p = 0.001): see Fig. 5. To consider the sex difference first, overall females with only one antenna were more aggressive than males with one antenna (W = 789.5, p = 0.001). This was the case also in female LR versus male LR (W = 97, p = 0.003). However, this sex difference was not apparent in dyads of bees with both antennae, BB, in which condition aggression was relatively low in all dyads.

Aggressive contacts were lateralized. As can be seen in Fig. 5, there were much higher scores of aggression in females in LL dyads than in RR dyads (W = 99.5, p = 0.002), and also significantly higher than in dyads of intact females, BB (W = 99.5, p = 0.0002).

Aggressive contacts were also elevated in LR dyads of females. These scores were significantly higher than in dyads of intact females, BB (W = 99.5, p = 0.0002). The difference between female LL and female LR dyads was not significant (W = 73.5, p = 0.203) and nor was the difference between female LR dyads and female RR dyads (W = 87, p = 0.0857).

In male-male dyads, aggression in LL was significantly higher than in RR (W = 93, p = 0.030) but the effect was not as marked as in females (see Fig. 5). Differing from females, aggressive contacts were not elevated in male LR dyads: the difference between female LR and male LR dyads was significant (W = 97, p = 0.0032). In males, aggressive contacts in LL were significantly higher than in BB (W = 97, p = 0.014) and in LL versus LR the p-value was p = 0.057 (W = 89).

In dyads of male + female, there was no left-right asymmetry and no difference between groups (Fig. 5).

### Bouts of wiping behaviour

**Figure 6:**
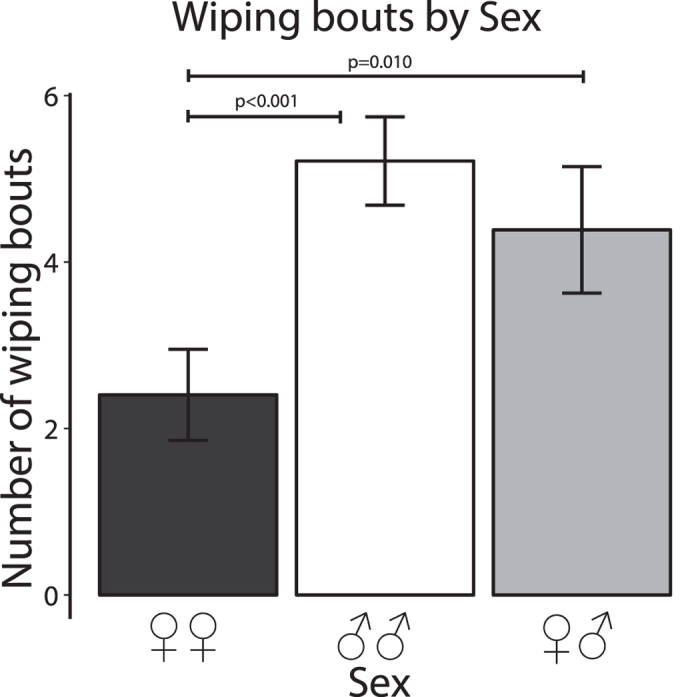
Bouts of wiping behaviour of the head or abdomen. The data are presented as in Fig. 3. Note the lower scores in female dyads.

Analysis of wiping behaviour, a self-directed individual behaviour, revealed no significant effect of Dyad (Kruskal-Wallis chi-squared_3_ = 5.543, p = 0.136) but a significant effect of Sex (Kruskal-Wallis chi-squared_2_ = 19.053, p < 0.001): see Fig. 6. The occurrence of wiping behaviour was lower in female-female dyads, regardless of antennal condition, than in male-male and female-male dyads (i.e., in B + B, L + L and R + R): female-female *vs.* male-male, W = 405.5, p < 0.001; female-female *vs*. female-male, W = 423, p = 0.010; male-male *vs.* female-male, W = 778.5, p = 0.155.

### Proportion of time in the same dish

**Figure 7:**
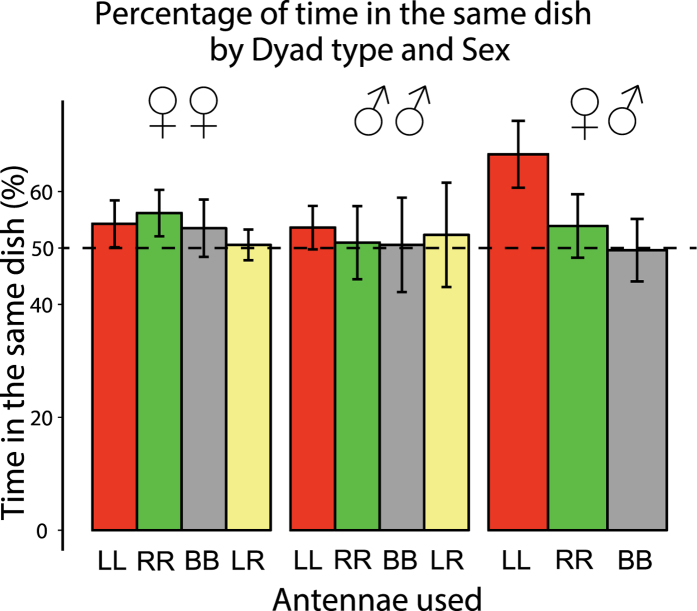
Percentage of time spent in the same dish. The bars are coloured in the same way as in Fig. 5. Note the higher scores of male + female dyads with left antennae (LL, red bars) in use.

These data were normally distributed (Shapiro test, W = 0.980, p = 0.07) and there was no significant main effect of Dyad type (F_3,104_ = 0.293, p = 0.83) or Sex (F_2,104_ = 1.125, p = 0.33) or interaction (F_5,104_ = 0.69, p = 0.63): see Fig. 7. Overall the dyads of our sample spent significantly more time in the same dish than expected by chance, as revealed by a one-sample t-test against chance level (0.5) (*t*_114_ = 2.24, p = 0.027) but the preference to be in the same dish was only mild (54% of the total time). Visual inspection of the data revealed that the male + female dyads with left antennae (LL) spent more time together than any other dyad (mean of 67%). Contrasting male + female LL dyads against all other dyads we observed a trend for more time spent together in these dyads but the difference was not quite statistically significant (t_16.99_ = −2.08, p = 0.053; p-value not corrected for multiple comparisons).

### Number of approaches by L and R bees in L + R dyads

In L + R dyads of females, the contacts made by L approaching R did not differ significantly from those made by R approaching L, regardless of whether the contact was non-aggressive (2-tailed paired test, t_10_ = 0.821, p = 0.431) or aggressive (t_10_ = −1.870, p = 0.091). Male L + R dyads made fewer contacts (see above) than did their equivalent female dyads and again there was no difference between contacts made by L approaching R or vice versa (for non-aggressive contacts, t_9_ = −1.354, p = 0.209; for aggressive contacts, t_9_ = −0.958, p = 0.363).

## Discussion

Lateralization was found in aggressive contacts. Scores of aggressive contacts, in which one bee rammed the other and caused the latter to adopt chaotic circling behaviour on its back and with buzzing, were significantly higher in dyads of bees with their left antennae only (LL). This asymmetry was present in both males and females, although it was stronger in females than in males, likely because females of this species are more aggressive than males^22^. Aggressive contacts were also elevated above control (both antennae intact, BB) levels in dyads of females in which one bee had a left antenna and the other a right antenna (LR) but not in the equivalent dyads of males. This result suggests that, in LR dyads of females, the female with its left antenna has a dominant role in social interactions, whereas in LR dyads of males the male with its right antenna assumes this dominant role. In females, the presence of just one bee using its left antenna only (i.e., in LR dyads) is sufficient to elevate aggressive interactions, although both aggressive and non-aggressive contacts were equally likely to be initiated by the L or R bee.

The chaotic buzzing behaviour following an aggressive interaction appeared to be an attempt to escape attack, although it did occur sometimes in other contexts.

Since *Osmia spp*. females defend their burrows to prevent them being usurped by other females^22^, we expected to see more agonistic behaviour in dyads of intact (BB) females than males. Furthermore, observations of wild mason bees report that males do not engage in direct social competition^26^. However, no such sex difference was evident in the testing conditions that we used. In dyads of bees using both antennae (BB), aggressive interactions were low in both males and females. It is possible that, when the bees could use either their left or right antenna, or both antennae, inputs from the right antenna drive behaviour and as a consequence aggression is inhibited. Aggressive contacts were elevated only when bees were forced to use the left antenna because the right antenna had been removed. Previous research has shown that laterality in responses driven by the antennae is not due to left-right differences in the number of antennal receptors in mason bees^29^. This suggests that the behavioural asymmetry in this species is not related to an asymmetry of the peripheral circuits of the antennae but it is more likely due to a different functional specialization of the corresponding side of the brain.

We did see an overall sex difference in the number of non-aggressive contacts: the scores were significantly higher in females overall than in males. It is notable that no lateralization was apparent in scores of the number of non-aggressive contacts. Therefore, asymmetric bias for higher aggressive behaviour when the left antenna is in use is a specific asymmetry and not merely an artefact of a left-right difference in number of contacts generally. Furthermore, it was not related in any way to differences in latency to the first contact or the amount of time spent in the same dish. Females made their first contact after shorter latency than did males but there was no significant difference between dyads with different antennae in use. Although there were no significant effects of sex or dyad type on scores for time spent in the same dish, we observed that dyads of one male and one female both with the left antenna in use were more likely to spend time together in the same dish than any other dyad. This suggests a role of functional lateralization for dyadic non-aggressive interactions between sexes, which could be a specialization for sexual behaviour.

The other sex difference that we observed was a reduced incidence in females of head or abdomen wiping with the legs. Since the wiping bouts, of the abdomen at least, may have involved release of pheromone, we suspect that this difference might be due to a sex difference in pheromone release but further experiments will be necessary to test this.

It is interesting that dyads of male + female performed in general more similarly to male + male dyads than to female + female dyads. Females were more active in contacting other females than they were in contacting males, which supports the observation in wild mason bees that agonistic social behaviour is more actively performed between females.

Our finding of lateralized antennal use in aggressive interactions between mason bees is important in comparison to the absence, in this species, of antennal laterality in olfactory memory recall and the absence of laterality in electroantennographic responses to particular odours^16^. Hence, we can conclude that mason bees show population-level lateralized antennal responses specifically when they engage in agonistic social behaviour. Given that the lateralization is observed in a specific functional context (aggressive interactions), it is most likely that it is a result of “higher” neuronal processes and is not simply induced by any peripheral asymmetry between the left and right antennae.

The presence of population-level asymmetries of motor performance in aggressive displays of so-called solitary insects, such as tephritid flies^27^ and mosquitoes^28^ may be explained in the same way as that of mason bees. Similar to mason bees, these flies have limited social behaviour but in both examples population-level lateralization is manifested in social competition.

This leads us to refine the original hypothesis, which predicted that population-level lateralization would be present in social but not non-social species^7^. Mason bees have limited social behaviour but they do exhibit directional laterality when they engage in that social behaviour. Competition between females for nest sites and between males for access to females would provide a context for aggressive behaviour and each antenna has a specialized role in this behaviour. Use of the left antenna stimulates aggression towards competitors and, possibly, attraction towards members of the opposite sex, and use of the right antenna suppresses these behaviours. Our results highlight the role of social demands in the evolution of population-level functional asymmetry and they suggest that not only does general sociality generate directional laterality but also that it involves engagement in specific social interactions. This may support the idea that the pattern of neuronal lateralization does not develop as a mere left-right dichotomy but depends on the specific requirements of different functions. Our findings could be interpreted as an example of dynamic regulation of behavioural asymmetry within the social context, meaning that lateralization is not necessarily a static feature of neuronal organization but it is modulated by the functional context.

## Methods

### Subjects

Cocoons of *Osmia bicornis* (Megachilidae) were obtained from WAB - Mauerbienenzucht, Germany, in early May, 2015. After they had been delivered to the laboratory, they were kept at 2 °C for 2 days until just before the experiments were to commence. At this time they were moved to insect tents at 25 °C and 60% humidity, separate ones for males and females. Hatching from the egg capsules began a few hours later and continued for 2 days in the case of males and for 7 days in the case of females. The tents were furbished with pots of flowering marigold plants and small roses, on which the bees could feed. They were also supplied with honeybee pollen (brand, Cuor di Miele, Italy) and 50% sucrose solution. The latter was presented as a shallow solution in Petri dishes or on crumpled absorbent paper.

Testing began as soon as the bees started to hatch and continued for two weeks. The bees were tested in dyads of the following combinations: two males, or two females, with both antennae intact (BB), both using the left antenna only (LL), both using the right antenna only (RR) or one using the left antenna and the other using the right antenna (LR). Male plus female dyads were also tested in the following combinations: both antennae intact (BB), both using the left antenna (LL) or both using the right antenna (RR). There were a total of 11 different types of dyad and between 10 and 12 dyads of each type. A total of 115 dyads was tested. Use of one antenna was achieved by removing the other antenna just prior to testing. The bee was held gently and briefly between the experimenter’s gloved fingers, during which time one antenna was sectioned at segment 1 or 2. Neither of these segments have sensillae, judging by research on *Osmia cornuta*^29^. Vannas micro-scissors (0.5 mm tips) were used to cut the antennae. Wearing rubber gloves largely prevented the females from stinging the experimenter. Males of this species do not sting. Once the antenna had been removed, the bee was placed in the testing apparatus. Intact bees were held between the fingers in the same manner but no antenna was cut.

### Testing apparatus

The apparatus was essentially the same as that used to test social interactions between honeybees and described in^23^. Two aerated Petri dishes (9 cm diameter and 1.5 cm in depth) were placed upside down and side-by-side. At the place of contact the lids had small openings (0.7 cm × 1.3 cm) and they were secured by clear adhesive tape on the underside, touching the floor. A similar opening was cut into the upper (previously the lower) lid of each dish so that, when all openings were aligned, the bees could move freely between the dishes. The bees were prevented from escaping from the apparatus by a small, clear plastic arch over the region of the adjoining openings. By turning the upper lids so that the openings were no longer aligned, the bee’s access from one dish to the other was prevented. Small pieces of clear plastic (approx. 1 cm squared) were slipped into place next to each opening to ensure that the bees could not escape when the openings were not aligned. This apparatus was placed inside a light blue bowl (26 cm diameter × 15 cm depth) with white washable plastic on the bottom. A web-cam (Lifecam Studio) placed directly above the dishes recorded the behaviour of the bees.

### Behavioural testing

Testing commenced with the upper dishes rotated so that their openings were blocked. A bee was placed in each of the dishes and remained thus for 5 minutes. Then the upper lids were rotated so that the openings were aligned and a further 10 minutes of testing took place. The bees in their Petri dishes were video-recorded.

In the 10-minute testing session (openings aligned) the following behaviour was scored from the videos: (1) latency to first physical contact, (2) number of physical contacts not involving high aggression (here referred to as ‘contacts’), (3) number of highly aggressive interactions, in which one bee rammed the other causing the latter to overturn followed by buzzing and moving in a “frantic” and chaotic manner (here referred to as ‘aggressive interactions’), (4) bouts of wiping, in which either of the bees wiped its abdomen or its head with the hind- or fore-legs, respectively, (5) time during which both bees were present in the same dish as a proportion of the total time recorded during a test – bees were scored as being in the same dish when (a) both bees were entirely in the same dish, (b) one had at least its head in the same dish as the bee or (c) when both bees spent at least 5 seconds in contact in the region connecting the two dishes, (6) in LR dyads the number of approaches made by the L bee to the R bee and *vice versa* in both non-aggressive contacts and aggressive contacts.

Inter-rater reliability was assessed by comparing the scores of two experimenters (LJR and EF) for all but number (5), in which case two independent observers, blind to the experimental hypotheses, scored. Considering 12 dyads selected at random across treatments, the following correlations between the two experimenters’ scores were as follows: r = 0.998 for latency, r = 0.925 for contacts and r = 0.911 for aggressive contacts. For the scores of number (5) data recorded from 73 bees were recorded by two observers and the correlation between observers was r = 0.922.

### Data analysis

Each type of behaviour measured was analysed separately. First, the data were checked for normality (Shapiro test) and in the case of latency they were normalised by log_10_ transformation. Hence, latency scores could be analysed by ANOVA using the factors Dyad type (LL, RR, BB and LR) and Sex (2 females, 2 males, male + female). The scores for non-aggressive contacts and proportion of time in the same dish were normally distributed and did not need to be transformed. *Post hoc* Tukey tests or two-tailed Welch-corrected t-tests were applied. Since the other data could not be normalised they were analysed using the non-parametric one-way test Kruskal Wallis for the factors Dyad-type and Sex. If significance was found, this analysis was followed by *post hoc* two-tailed Wilcoxon tests.

## Additional Information

**How to cite this article**: Rogers, L. J. *et al*. Lateralized antennal control of aggression and sex differences in red mason bees, *Osmia bicornis*. *Sci. Rep.* **6**, 29411; doi: 10.1038/srep29411 (2016).

## Figures and Tables

**Figure 1:**
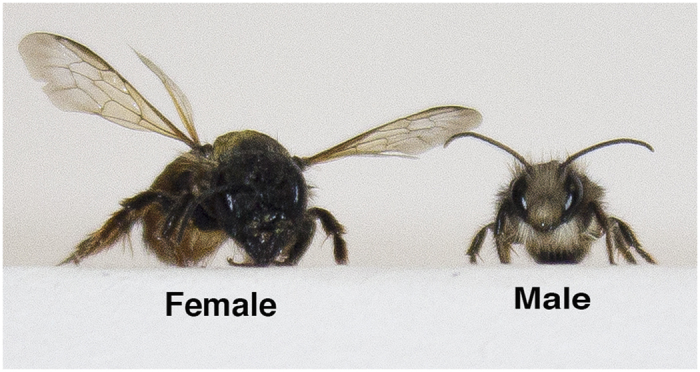
Photograph of a female and a male mason bee. Note the size difference and the longer antennae in the male. Photograph by M.L.C. (Nia) Iurilli.
